# Comparison of consumption behavior and appetite sensations among patients with type 2 diabetes mellitus after bariatric surgery

**DOI:** 10.7717/peerj.3090

**Published:** 2017-03-21

**Authors:** Chun Yeh, Hsien-Hao Huang, Shu-Chun Chen, Tung-Fang Chen, Kong-Han Ser, Chih-Yen Chen

**Affiliations:** 1 Division of Gastroenterology, Department of Internal Medicine, Cheng-Hsin General Hospital, Taipei, Taiwan; 2 Department of Emergency Medicine, Taipei Veterans General Hospital, Taipei, Taiwan; 3 Institute of Emergency and Critical Medicine, National Yang-Ming University School of Medicine, Taipei, Taiwan; 4 Department of Nursing, Min-Sheng General Hospital, Taoyuan, Taiwan; 5 Taiwan Society for Metabolic and Bariatric Surgery, Taoyuan, Taiwan; 6 Medical Affairs Office, Taipei City Hospital Yangming Branch, Taipei, Taiwan; 7 Department of Surgery, Min-Sheng General Hospital, Taoyuan, Taiwan; 8 Division of Gastroenterology and Hepatology, Department of Medicine, Taipei Veterans General Hospital, Taipei, Taiwan; 9 Faculty of Medicine, National Yang-Ming University School of Medicine, Taipei, Taiwan; 10 Taiwan Association for the Study of Small Intestinal Diseases, Guishan, Taiwan

**Keywords:** Appetite sensation, Bariatric surgery, C-peptide, Mixed meal tolerance test, Sleeve gastrectomy, Diabetes mellitus, Gastric bypass

## Abstract

**Background:**

The promising postsurgical weight loss and remission of type 2 diabetes (T2D) from bariatric surgery can be attributed to modified eating physiology after surgical procedures. We sought to investigate the changes in the parameters of consumption behaviors and appetite sensations induced by a mixed meal tolerance test, and to correlate these alterations with age, body mass index, C-peptide levels, and duration of T2D 1 year after bariatric surgery.

**Methods:**

A total of 16 obese patients with T2D who underwent mini-gastric bypass (GB) and 16 patients who underwent sleeve gastrectomy (SG) were enrolled in this study and evaluated using a mixed meal tolerance test one year after surgery. A visual analogue scale was used for scoring appetite sensation at different time points. The area under the curve (AUC) and the incremental or decremental AUC (ΔAUC) were compared between the two groups.

**Results:**

One year after surgery, a decreasing trend in the consumption time was observed in the GB group compared to the SG group, while the duration of T2D before surgery was negatively correlated with the post-operative consumed time in those after GB. Patients who underwent GB had significantly higher fasting scores for fullness and desire to eat, higher AUC_0′–180′_ of scores for desire to eat, as well as more effective post-meal suppression of hunger and desire to eat compared with those undergoing SG one year after surgery. Post-operative C-peptide levels were negatively correlated with ΔAUC_0′–180′_ for hunger and ΔAUC_0′–180′_ for desire to eat in the GB group, while negatively correlated with ΔAUC_0′–180′_ for fullness in the SG group.

**Discussion:**

Patients with T2D after either GB or SG exhibit distinct nutrient-induced consumption behaviors and appetite sensations post-operatively, which may account for the differential effects on weight loss and glycemic control after different surgery.

## Introduction

The rapidly increasing incidence of obesity is emerging as a worldwide public health problem. As the prevalence of obesity continues to increase, the incidence of diabetes is propelled to upsurge (Wild et al., 2004; Hossain, Kawar & El Nahas, 2007). The rate of attribution of type 2 diabetes (T2D) to obesity is approximately 90% (Isomaa et al., 2001). Bariatric surgery, including gastric bypass (GB) and sleeve gastrectomy (SG), is known as the most effective method of improving obesity, and it is a potential strategy for remission of T2D (Schauer et al., 2014). The promising postsurgical weight loss and the maintenance of weight loss might be a result of modified eating behavior (Saunders, 1999; Laurenius et al., 2012) or it may be directly due to the surgical procedure (Saunders, 2001). GB and SG have been shown to effectively increase satiety sensation (Valderas et al., 2010). Good control of satiety might be related to the attenuation of insulin response (Speechly & Buffenstein, 1999), while intranasal insulin has been shown to reduce the stimulation of brain activity caused by pictures of food (Guthoff et al., 2010). In addition, intracerebroventricular administration of insulin significantly inhibited food intake and functions as an appetite-suppressive peptide (Chen et al., 2012). Bariatric surgery has been demonstrated to reduce C-peptide levels and improve insulin resistance (Lee et al., 2013). The improvement of diabetes after bariatric surgery is due to the up-regulation of the insulin signaling pathway (Bonhomme et al., 2011).

C-peptide, a 31 amino-acid polypeptide, is considered to be a marker for pancreatic insulin secretion (Bonser & Garcia-Webb, 1984; Hovorka & Jones, 1994). Our previous studies proposed a diabetes surgery score, the ABCD score (age, body mass index, C-peptide and duration of T2D), which is a clinically applicable parameters used to predict the success of bariatric surgery in obese patients with T2D (Lee et al., 2013; Lee et al., 2015). Diabetic patients with higher ABCD score before surgery had a higher rate of T2D remission (Lee et al., 2013; Lee et al., 2015). However, the relationships between ABCD score with consumption behaviors and appetite sensations after bariatric surgery are still unknown. In this study, we used the mixed meal tolerance test (MMTT) to investigate the changes in consumption behaviors and appetite sensations, and we correlated these alterations with age, body mass index, C-peptide, and duration of T2D one year after bariatric surgery.

## Methods

### Patients and metabolic surgery

The patients were enrolled in a previous randomized trial, which included 32 eligible patients with T2D (laparoscopic mini-gastric bypass (GB), *N* = 16; SG, *N* = 16). The GB (Lee et al., 2011b) and SG surgery (Lee et al., 2011b) were performed as previously described. One year after bariatric surgery, all 32 patients agreed to undergo the MMTT. Informed consent was obtained before the study.

This study was conducted at the Department of Surgery of Min-Sheng General Hospital and at Taipei Veterans General Hospital, and was approved by the Ethics Committee of each hospital (the approval number: 201002056IC and 201002037IC).

### Surgical technique

GB was performed as described in our previous studies (Lee et al., 2011b; Lee et al., 2011a). In brief, we used a standard 5-port laparoscopic technique to create a long-sleeve gastric tube by using the EndoGIA stapler (EndoGIA; Coviden, Norvalk, CT, USA); it was approximately 2.0 cm wide along with the lesser curvature from the antrum to the angle of His. We also use an EndoGIA stapler to create a Billroth II type loop gastroenterostomy with the small bowel about 120 cm distal to the ligament of Treitz. There was no drain tube left in place. By using the mesh plug technique with bio-absorbable hemostatic gauze (Cellulostat; Horng Tzer Medical Instruments, Kaohsiung, Taiwan) (Chiu et al., 2006), we closed all the trocar wounds.

For SG, we used a laparoscopic stapler (EndoGIA; Coviden, Norvalk, Connecticut) with 60-cm cartridges (3.5 mm stapler height, blue load) to resect the greater curvature from the distal antrum (4 cm proximal to the pylorus) to the angle of His, including the complete fundus (Lee et al., 2011b). We left the remnant stomach tube, which was approximately 2 cm wide along the less curved side. The extended periumbilical trocar site was used for the extraction of the resected stomach portion.

### Mixed meal tolerance test

To compare the consumption behavior and appetite sensations among patients with T2D after metabolic surgery, we used an MMTT. After an overnight fast, patients ate ensure plus (4 oz; 175 kcal; 6.5 g protein; 5.5 g fat; 25 g carbohydrate; Abbott Nutrition, Zwolle, Netherlands) (Lee et al., 2011a). The volume consumed was recorded and the consumed energy was calculated based on the volume of the MMTT after digestion.

### Questionnaire on hunger, fullness, desire to eat, satiation, and prospective consumption

A visual analogue scale (VAS) of 100-mm lines was designed for patients to score their appetite sensations after a test meal with good reliability and validity (Parker et al., 2004). Similar to our previous study (Chen et al., 2013; Zachariah et al., 2016), the participants were familiarized with assessing the VAS and scoring their appetite sensations, such as hunger, fullness, desire to eat, satiation, and prospective consumption before food intake (0 min) and at 30, 60, 90, 120, 150 and 180 min after a mixed meal. The area under the curve (AUC) and the incremental or decremental area under the curve (ΔAUC) of various appetite VAS scores during the MMTT were measured by the trapezoidal method (He et al., 2011; Lee et al., 2011a). The VAS score for peak-0′ was calculated by subtracting the score at 0 min (before food intake) from the highest score recorded during the MMTT. The VAS score for peak-nadir was calculated by subtracting the lowest score from the highest score recorded during the MMTT.

## Statistical analysis

All statistical analyses were performed using the Statistical Package for Social Sciences, version 12.01 (SPSS, Inc., Chicago, IL, USA). Continuous variables were expressed as the mean ± SD. The chi-square test or Fisher’s exact test was used to compare categorical variables, while the Mann–Whitney *U* test was used to compare continuous variables. The Wilcoxon signed-rank test was used to compare between baseline and post-operative variables. Friedman’s one-way analysis of variance followed by a *post hoc* test was used to analyze the differences among VAS scores for different appetite sensations at 0, 30, 60, 90, 120, 150, and 180 min after intake of a mixed meal. Correlations between the two groups were examined using Spearman’s correlation method. A *P* value less than 0.05 was considered statistically significant.

## Results

### Treatment effect one year after bariatric surgery

In total, 16 patients undergoing GB and 16 patients undergoing SG were enrolled. Before surgery, there are no static significant differences between GB and SG in age, BMI, C-peptide, the duration of T2D, and homeostasis model assessment-insulin resistance (HOMA-IR) (Table 1). One year after bariatric surgery, the BMI in the GB and SG groups were 22.6 ± 2.5 and 24.4 ± 2.5 kg/m^2^ (*P* < 0.05), respectively, which was the only statistically significant difference between the two groups. Although the BMI was lower in patients after GB than those after SG, but the ratio of BMI after/before either GB or SG had no significant difference (*P* > 0.05). Post-operative levels of C-peptide and HOMA-IR were comparable between the GB and SG groups one year after surgery (Table 1).

**Table 1:** ABCD scale (age, body mass index, C-peptide, and duration of T2D) before and 1 year after bariatric surgery.

	GB (*n* = 16)	SG (*n* = 16)	*P* value
Age before surgery	44.3 ± 8.6	46.3 ± 8.0	NS
BMI before surgery	29.1 ± 3.1	31.0 ± 2.9	NS
BMI one year after surgery	22.6 ± 2.5	24.4 ± 2.5	<0.05
Ratio of BMI after/before surgery	0.78	0.79	NS
C-peptide before surgery	2.5 ± 1.1	3.3 ± 1.4	NS
C-peptide one year after surgery	1.6 ± 1.1	1.7 ± 0.5	NS
Duration of T2D before surgery	6.0 ± 5.5	6.9 ± 5.4	NS
HOMA-IR before surgery	9.3 ± 23.2	8.5 ± 6.5	NS
HOMA-IR one year after surgery	1.2 ± 1.2	2.4 ± 3.5	NS

**Notes.**

Data is expressed as the mean ± SD.

BMI body mass index HOMA-IR homeostasis model assessment-insulin resistance GB gastric bypass SG sleeve gastrectomy T2D type 2 diabetes

### The volume, energy, and time of consumption after bariatric surgery

**Figure 1:**
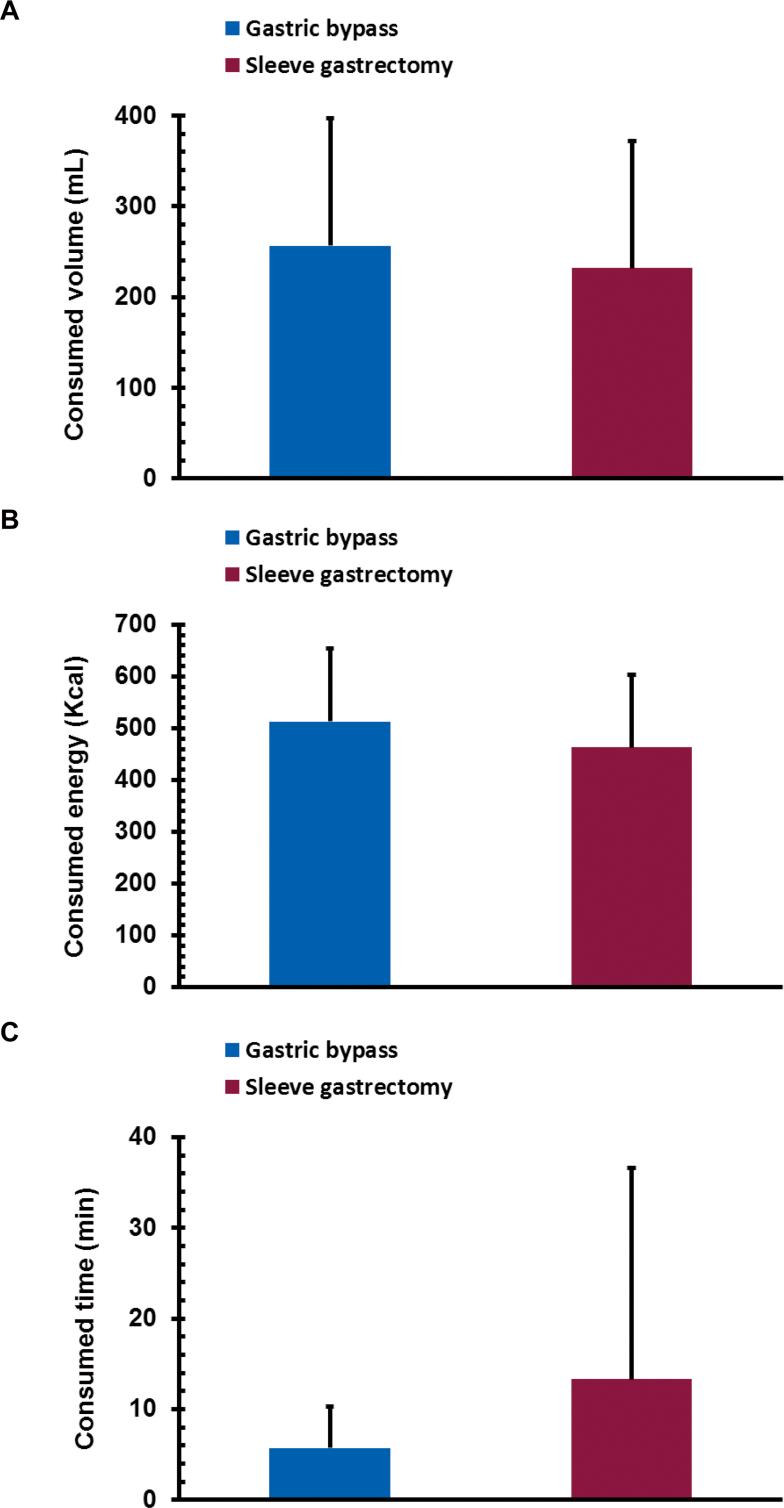
The volume of food consumed (A), energy consumed (B), and consumption time (C) during a mixed meal tolerance test in gastric bypass (*n* = 16) and sleeve gastrectomy (*n* = 16) patients one year after surgery. Data is expressed as the mean ± SD.

One year after surgery, there were no significant differences in the volume of consumption between the GB and SG groups (256.6 ± 142.3 mL vs. 231.8 ± 140.3 mL, *P* > 0.05; Fig. 1A). The total energy consumed in the GB and SG groups was comparable (513.1 ± 284.6 Kcal vs. 463.5 ± 280.5 Kcal, *P* > 0.05; Fig. 1B). The time of consumption in the GB had a decreasing trend compared to that in SG (5.7 ± 4.6 min vs. 13.3 ± 23.3 min, *P* > 0.05; Fig. 1C).

### Hunger sensation during the MMTT after bariatric surgery

**Figure 2:**
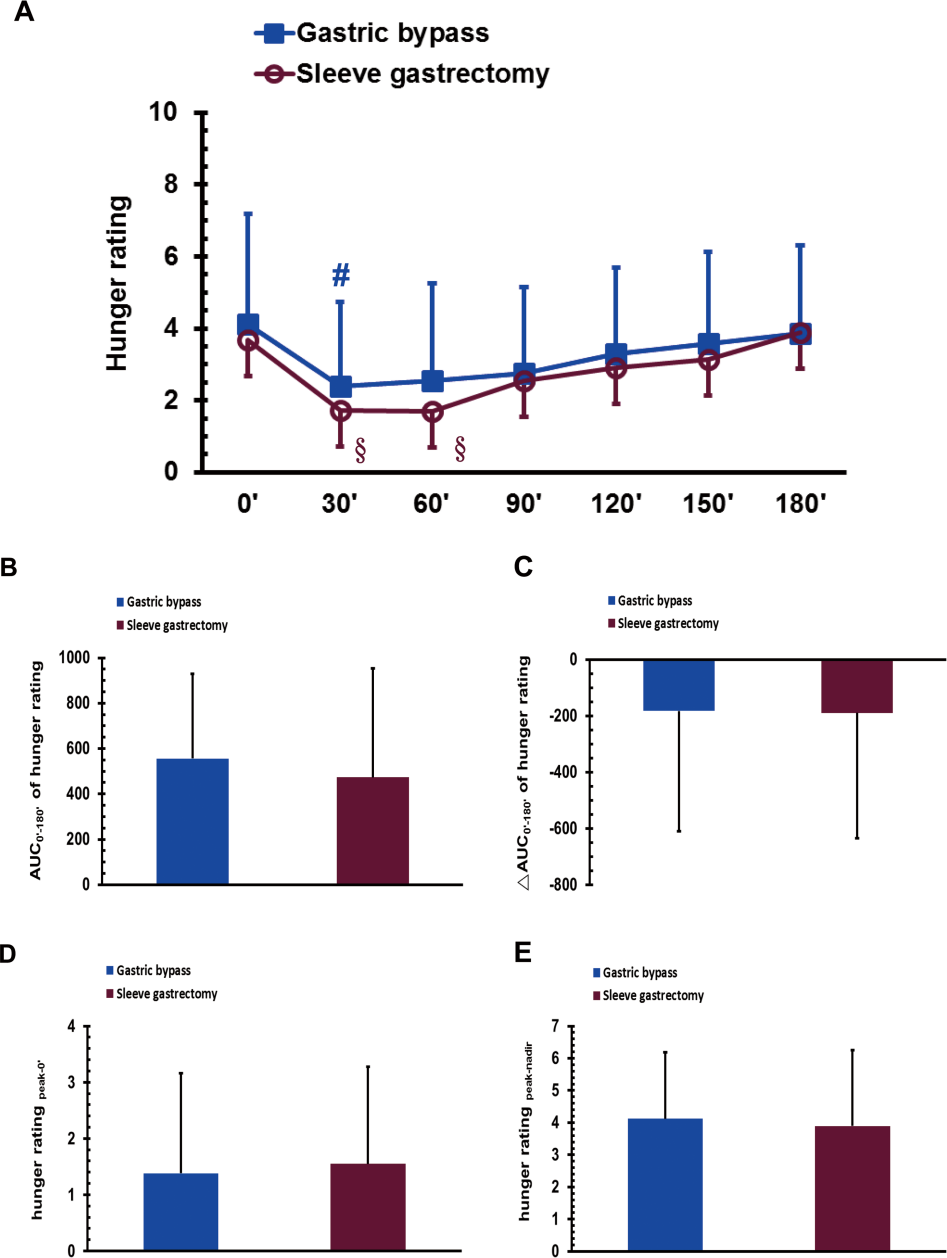
The visual analogue scale scores of hunger sensation in gastric bypass and sleeve gastrectomy patients at 0, 30, 60, 90, 120, 150 and 180 min (A), as well as the AUC_0′–180′_ of the hunger rating (B), the ΔAUC_0′–180′_ of the hunger rating (C), the hunger rating _peak-0′_ (D), and the hunger rating _peak-nadir_ (E) in a mixed meal tolerance test. # *P* < 0.05 compared to 0 min, §*P* < 0.05 compared to 180 min.

The VAS score of hunger sensation in the GB group, but not in the SG group, at 30 min was significantly lower than that at 0 min in the MMTT (*P* < 0.05; Fig. 2A). The scores for hunger sensation in the SG group at 30 and 60 min were significantly lower than that at 180 min (*P* < 0.05 and 0.05; Fig. 2A). No significant difference was found between the GB and SG groups for the AUC_0′–180′_ of the hunger score, the ΔAUC_0′–180′_ of the hunger score, the hunger score for _peak-0′_ or the hunger score for _peak-nadir_ (Figs. 2B–2E).

### Fullness sensation in MMTT after bariatric surgery

**Figure 3:**
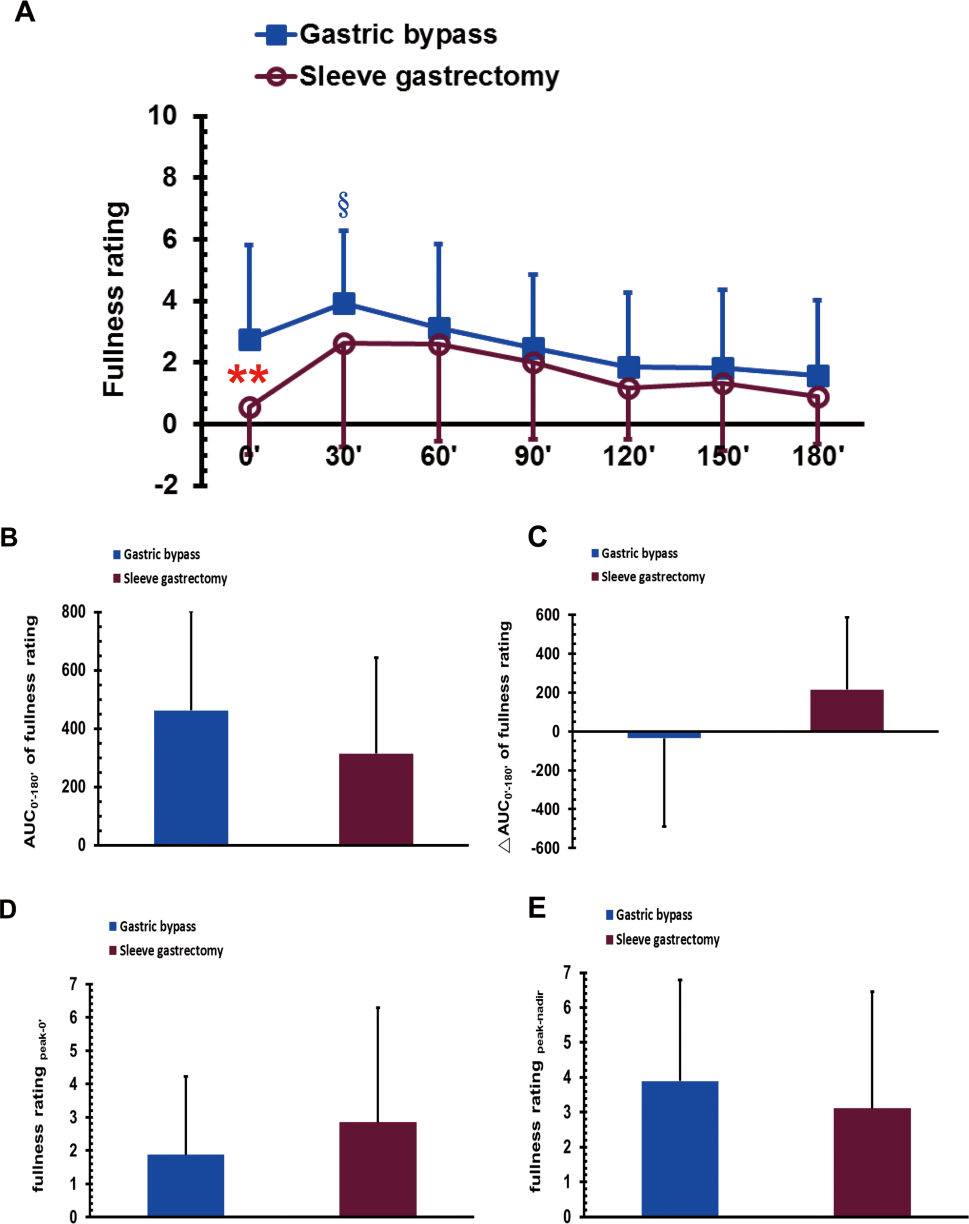
The visual analogue scale scores of fullness sensation in gastric bypass and sleeve gastrectomy patients at 0, 30, 60, 90, 120, 150 and 180 min (A), as well as the AUC_0′–180′_ of the fullness rating (B), the ΔAUC_0′–180′_ of the fullness rating (C), the fullness rating _peak-0′_ (D), and the fullness rating _peak-nadir_ (E) in a mixed meal tolerance test. ** *P* < 0.01 compared between gastric bypass and sleeve gastrectomy groups, § *P* < 0.05 compared to 120, 150, and 180 min.

The VAS score of postoperative fasting fullness sensation was significantly higher in the GB group than in the SG group (*P* < 0.01; Fig. 3A). The score for fullness sensation in the GB group at 30 min was significantly higher than that at 120, 150, and 180 min in the MMTT (*P* < 0.05; Fig. 3A). No significant difference was found between the GB and SG groups in either the AUC_0′–180′_ of the fullness rating, the ΔAUC_0′–180′_ of the fullness rating, the fullness score for _peak-0′_ or the fullness score for _peak-nadir_ (Figs. 3B–3E).

### Desire to eat sensation in the MMTT after bariatric surgery

**Figure 4:**
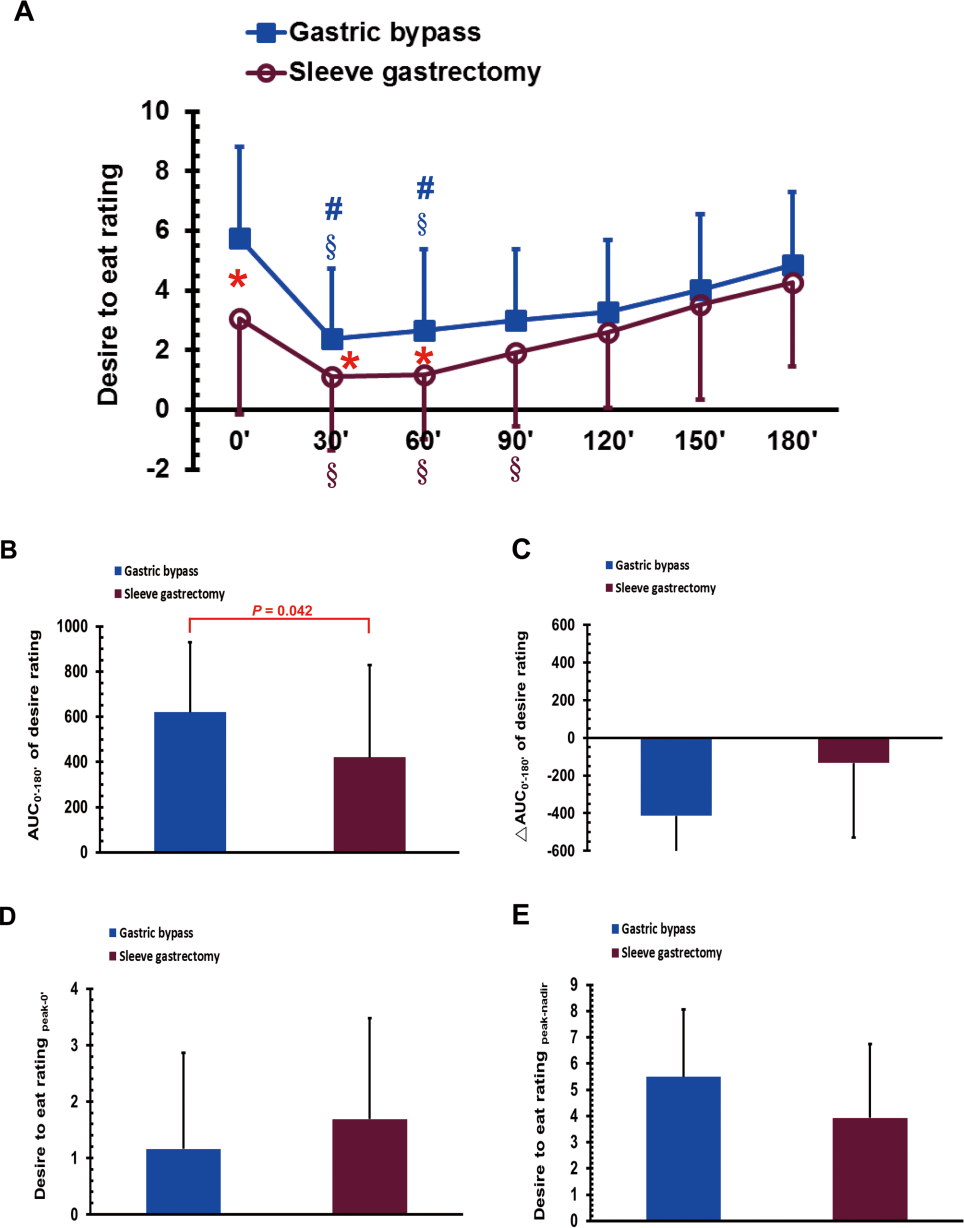
The visual analogue scale scores of desire to eat in gastric bypass and sleeve gastrectomy patients at 0, 30, 60, 90, 120, 150 and 180 min (A), as well as the AUC_0′–180′_ of the desire to eat (B), the ΔAUC_0′–180′_ of the desire to eat (C), the desire to eat_peak-0′_ (D) and the desire to eat _peak-nadir_ (E) in a mixed meal tolerance test. * *P* < 0.05 compared between gastric bypass and sleeve gastrectomy groups, # *P* < 0.05 compared to 0 min, §*P* < 0.05 compared to 180 min.

The VAS scores for postoperative desire to eat were significantly higher in the GB group than in the SG group at 0, 30, and 60 min (*P* < 0.05; Fig. 4A). The scores for desire to eat in the GB group at 30 and 60 min were significantly lower than that at 0 and 180 min during the MMTT (*P* < 0.05; Fig. 4A). The scores for desire to eat in the SG group at 30, 60, and 90 min were significantly lower than that at 180 min during the MMTT (*P* < 0.05; Fig. 4A). The AUC_0′–180′_ of the desire to eat was higher in the GB group than in the SG group (*P* < 0.05; Fig. 4B). No significant differences were observed between the GB and SG groups in either the ΔAUC_0′–180′_ of the desire to eat rating, the desire to eat rating for _peak-0′_, or the desire to eat rating for _peak-nadir_ (Figs. 4C–4E).

### Satiation sensation during the MMTT after bariatric surgery

**Figure 5:**
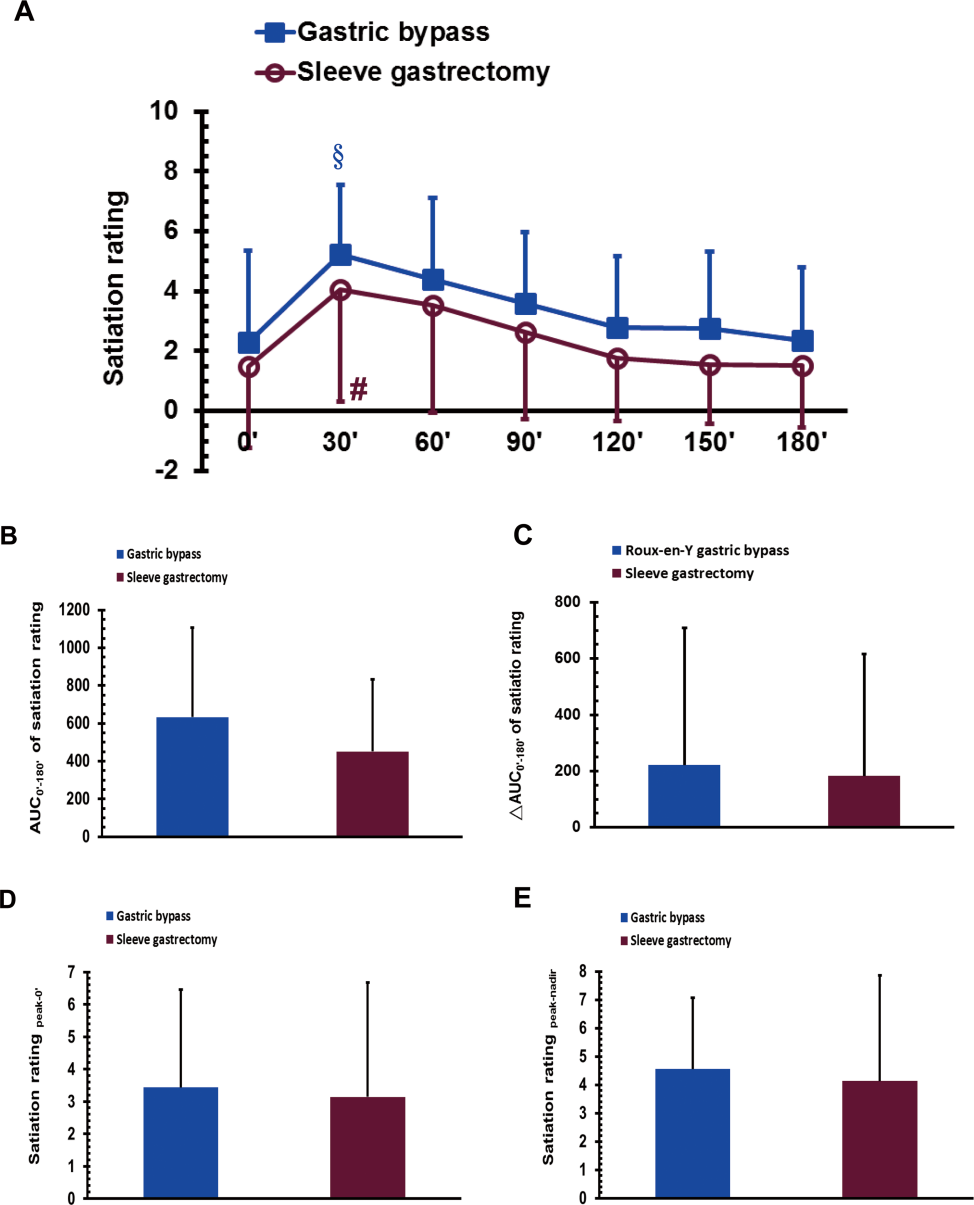
The visual analogue scale scores of satiation in gastric bypass and sleeve gastrectomy patients at 0, 30, 60, 90, 120, 150 and 180 min (A), as well as the AUC_0′–180′_ of satiation (B), the ΔAUC_0′–180′_ of satiation (C), the satiation _peak-0′_ (D), and the satiation _peak-nadir_ (E) in a mixed meal tolerance test. # *P* < 0.05 compared to 0 min, § *P* < 0.05 compared to 150 and 180 min.

The VAS score for satiation sensation in the GB group at 30 min was significantly higher than that at 150 and 180 min during the MMTT (*P* < 0.05; Fig. 5A). The VAS score for satiation in the SG at 30 min was significantly higher than that at 0 min during the MMTT (*P* < 0.05; Fig. 5A). No significant difference was observed between the GB and SG groups for either the AUC_0′–180′_ of satiation, the ΔAUC_0′–180′_ of satiation, the satiation score for _peak-0′_ or the satiation score for _peak-nadir_ (Figs. 5B–5E).

### Prospective consumption sensations during the MMTT after bariatric surgery

**Figure 6:**
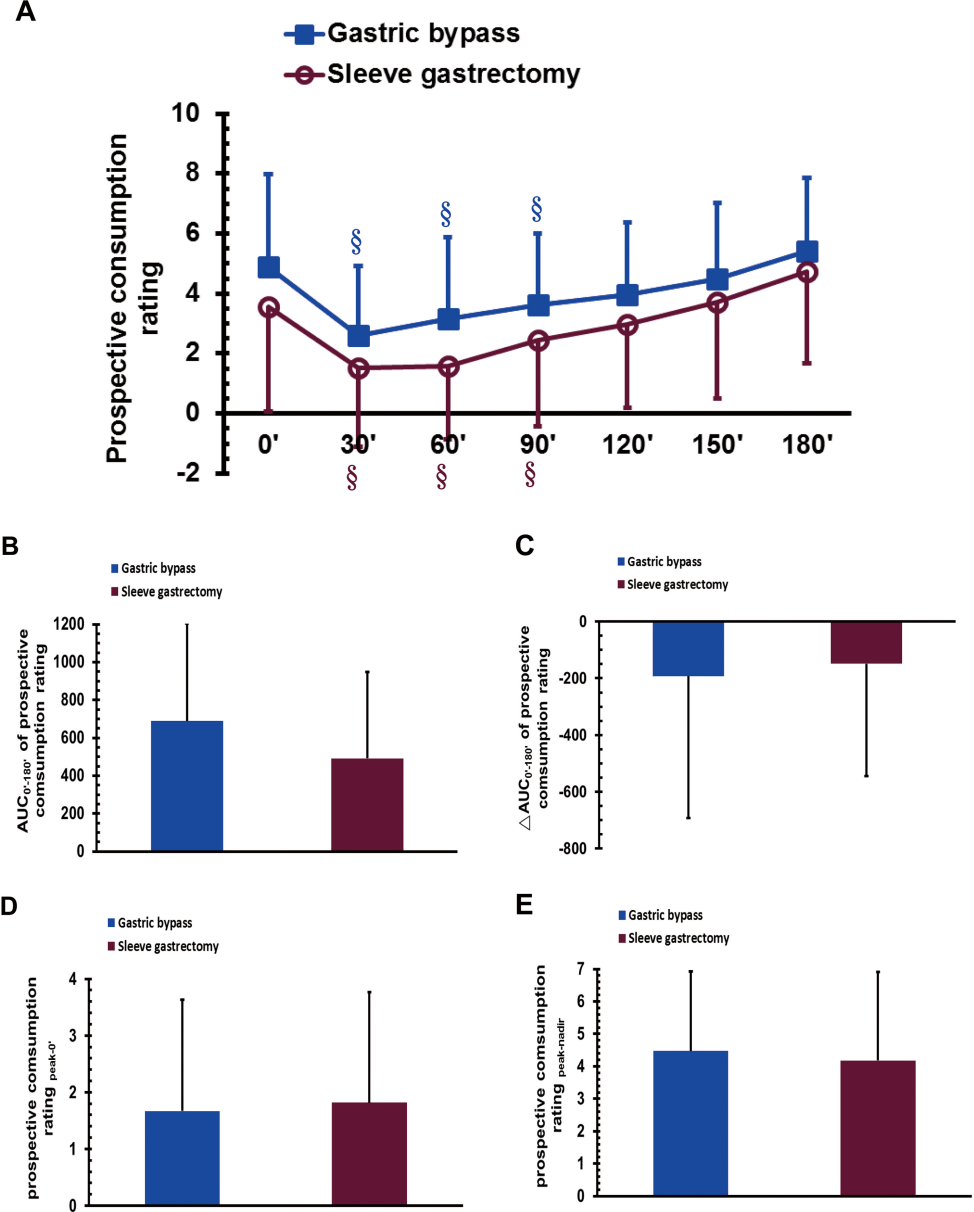
The visual analogue scale scores of prospective consumption in gastric bypass and sleeve gastrectomy patients at 0, 30, 60, 90, 120, 150 and 180 min (A), as well as the AUC_0′–180′_ of prospective consumption (B), the ΔAUC_0′–180′_ of prospective consumption (C), the prospective consumption _peak-0′_ (D), and the prospective consumption _peak-nadir_ (E) in a mixed meal tolerance test. §*P* < 0.05 compared to 180 min.

The VAS scores of prospective consumption in the GB group at 30, 60, and 90 min were significantly lower than that at 180 min during the MMTT (*P* < 0.05; Fig. 6A); a similar trend was observed for the SG group (*P* < 0.05; Fig. 6A). No significant difference was observed between the GB and SG groups in either the AUC_0′–180′_ of prospective consumption, the ΔAUC_0′–180′_ of prospective consumption, the prospective consumption score for _peak-0′_, or the prospective consumption score for _peak-nadir_ (Figs. 6B–6E).

### The correlations between ABCD score and consumption behaviors

In the GB group, the duration of T2D before surgery was negatively correlated with the post-operative consumed time during the MMTT (*ρ* =  − 0.617, *P* = 0.0105). The other parameters of ABCD score was not correlated with any consumption behavior.

In the SG group, there was no any correlation between any parameter of ABCD score and any consumption behavior.

### The correlations between ABCD score and appetite sensations

#### The correlations between the age or duration of T2D before surgery with appetite sensations

In the GB group, there was no any correlation between the age or duration of T2D before surgery, with any appetite sensations.

In the SG group, the age was negative correlated with the post-operative ΔAUC_0′–180′_ of the desire to eat rating (*ρ* =  − 0.569, *P* = 0.021), and the post-operative fasting satiation rating (*ρ* =  − 0.554, *P* = 0.026). In addition, the duration of T2D before surgery was positively correlated with the post-operative fasting satiation rating (*ρ* = 0.501, *P* = 0.048).

#### The correlations between BMI and appetite sensations

**Figure 7:**
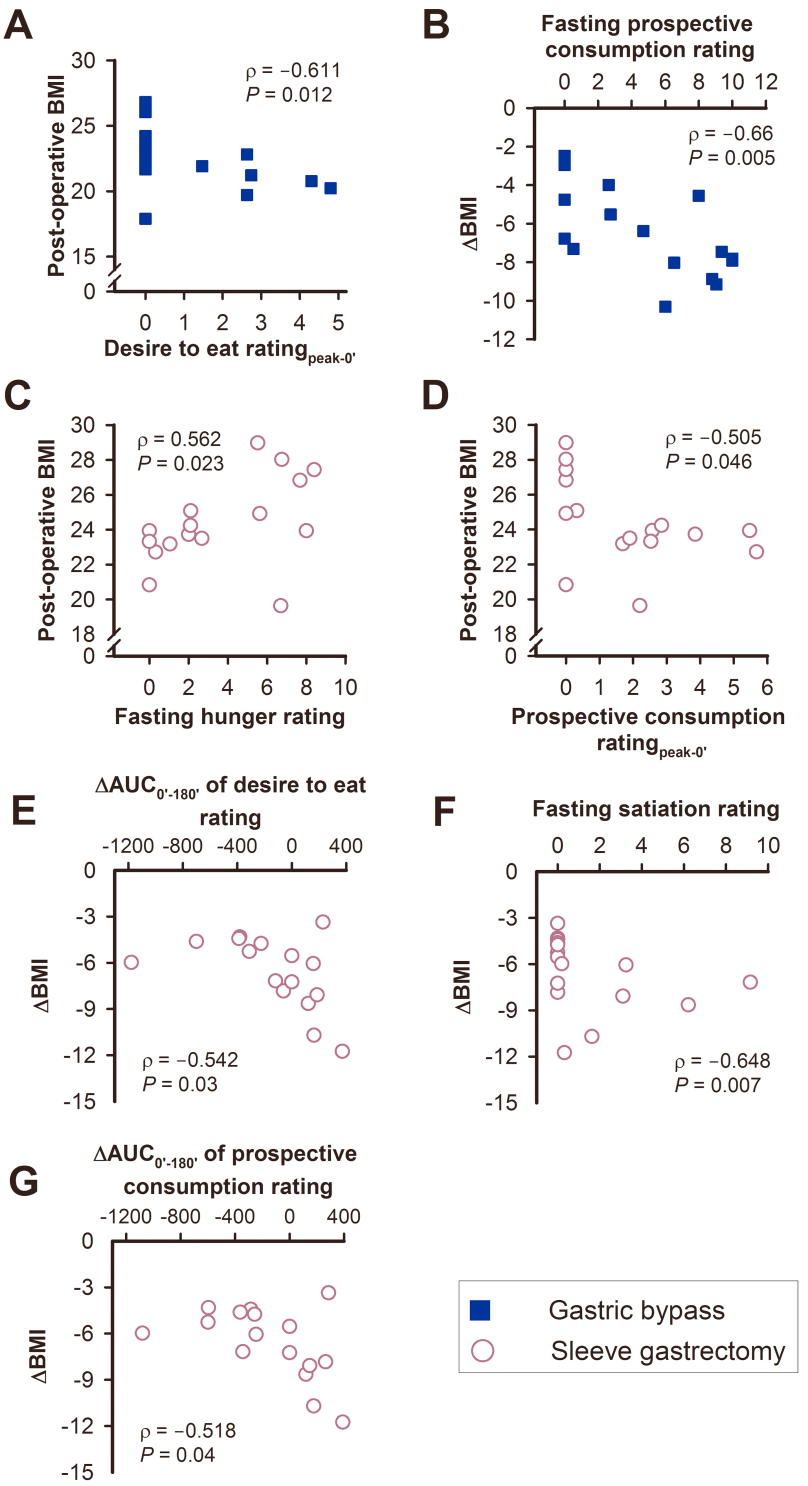
The relationships between post-operative BMI or ΔBMI with various visual analogue scale scores of appetite sensations in the gastric bypass (A, B) and sleeve gastrectomy (C–G) group.

In the GB group, post-operative BMI was negatively correlated with the desire to eat rating for _peak-0′_, while the ΔBMI was negatively correlated with post-operative fasting VAS scores for prospective consumption one year after surgery (Figs. 7A–7B).

In the SG group, post-operative BMI was positively correlated with the post-operative fasting score for hunger one year after surgery, and negatively correlated with the prospective consumption rating for _peak-0′_ (Figs. 7C–7D). The ΔBMI was negatively correlated with the post-operative ΔAUC_0′–180′_ of the desire to eat rating, the post-operative fasting score for satiation, and the post-operative ΔAUC_0′–180′_ of the prospective consumption (Figs. 7E–7G).

**Figure 8:**
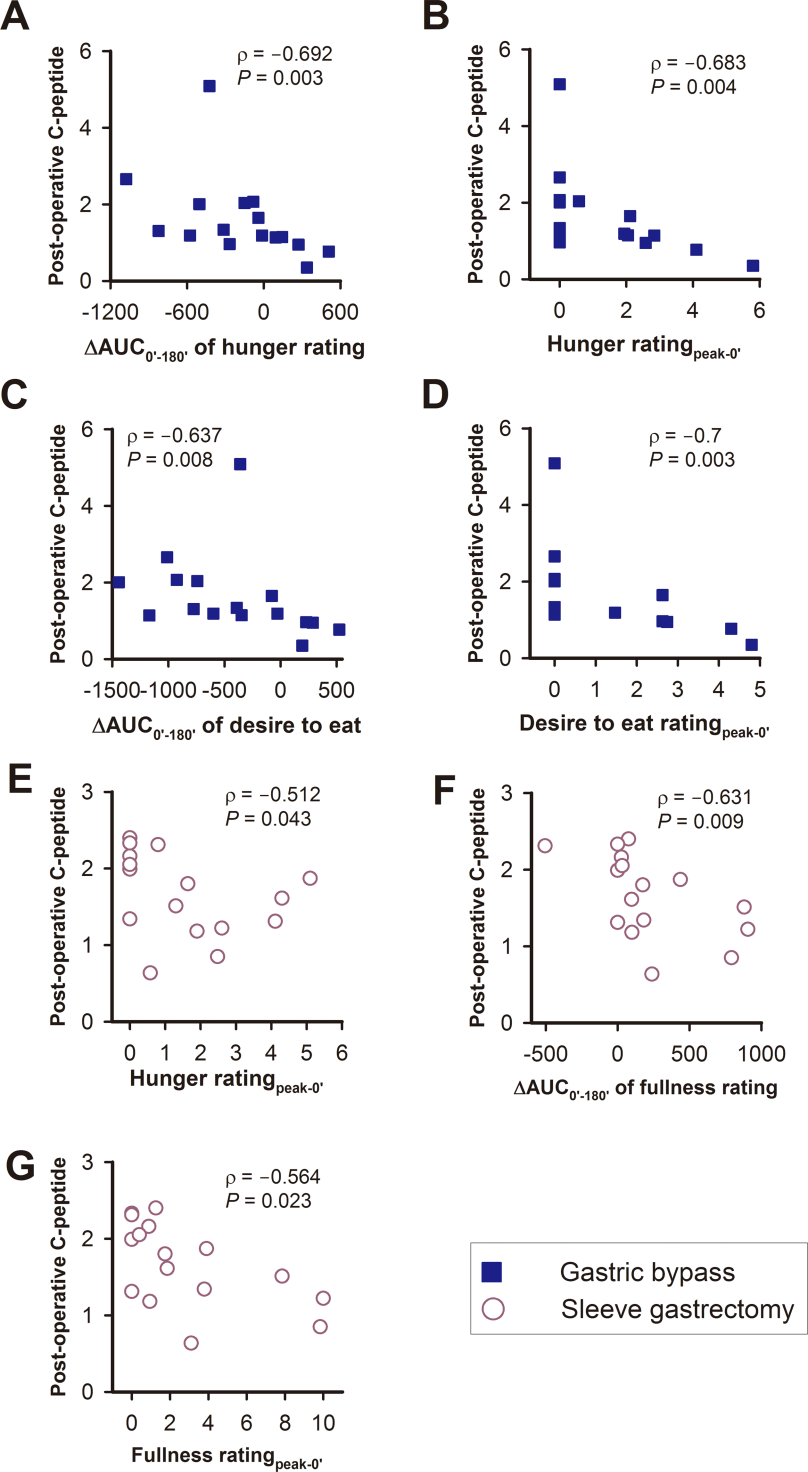
The relationships between post-operative C-peptide levels with various visual analogue scale scores of appetite sensations in the gastric bypass (A–D) and sleeve gastrectomy (E–G) group.

#### The correlations between C-peptide levels and appetite sensations

In the GB group, post-operative C-peptide levels were negatively correlated with the ΔAUC_0′–180′_ of the hunger rating, the hunger rating for _peak-0′_, ΔAUC_0′–180′_ of the desire to eat, and the desire to eat rating for _peak-0′_ (Figs. 8A–8D).

In the SG patients, post-operative C-peptide levels were negatively correlated with the hunger rating for _peak-0′_, ΔAUC_0′–180′_ of the fullness rating and the fullness rating score for _peak-0′_ (Figs. 8G–8H).

## Discussion

Bariatric surgery is a promising method for the treatment of obesity by maintaining long-term weight loss, improving risk factors, and decreasing overall mortality (Sjöström, 2008). After bariatric surgery, patients have lower energy intake and higher physical activity levels than obese patients without surgery (Sjöström et al., 2004). These changes might be attributed to modified eating behavior (Saunders, 1999; Laurenius et al., 2012) or bariatric surgical procedures (Saunders, 2001).

VAS scores for appetite are significantly attenuated after GB and SG (Karamanakos et al., 2008), and the volume of consumed food is consistently decreased after bariatric surgery (le Roux et al., 2006). Patients who underwent GB had decreased AUC_0′–180′_ of the scores for hunger sensation (Borg et al., 2006; Valderas et al., 2010) and increased AUC_0′–180′_ of the scores for satiety sensation (Korner et al., 2005; Borg et al., 2006; Valderas et al., 2010) as compared with obese patients who did not undergo surgery or with the preoperative condition. GB patients also had a greater increase in satiety sensations than gastric banding patients (Korner et al., 2006). In SG patients, satiety sensation was increased (Valderas et al., 2010), but the results for hunger sensation were inconsistent (Himpens, Dapri & Cadière, 2006; Valderas et al., 2010). Our previous study showed no significant difference in appetite sensation between patients treated with SG and those who underwent duodenal-jejunal bypass with SG (Zachariah et al., 2016).

In this study, we compared the consumption behaviors and appetite sensations of GB and SG patients one year after surgery. The ratio of BMI after/before surgery had no significant difference between GB and SG, which is comparable with our previous study (Lee et al., 2011a). The effect of GB on BMI is similar to that of SG. Interestingly, T2D patients after GB had a decreasing trend of length of time of food consumption compared to those after SG. Because ABCD score is a validated, simple multidimensional grading system to predict successful diabetes remission after GB (Lee et al., 2013) and SG (Lee et al., 2015), our further analysis revealed that the duration of T2D was negatively correlated with the post-operative consumed time during the MMTT in the GB but not in the SG group. This novel finding highlights the importance of the duration of T2D in determining consumption behaviors after GB, in addition to its prediction value in diabetes remission.

Patients who underwent GB had significantly higher fasting scores for fullness and desire to eat, higher AUC_0′–180′_ of desire to eat, as well as more effective post-meal suppression of hunger and desire to eat compared with those undergoing SG one year after surgery. Taken together, T2D patients after GB might initially have a higher desire to eat with an opposing pre-meal fullness, as well as more significant post-meal suppression of hunger at 30 min and desire to eat at 30 and 60 min during the MMTT compared with those after SG, resulting in a trend toward a shorter length of time of food consumption in patients receiving GB one year after surgery. These differences in appetite sensations between T2D patients after either GB or SG may result from the different surgical procedures. Further studies are required for a detailed explanation of this interesting phenomenon.

T2D remission rates of 93% and 47% can be achieved in GB and SG patients, respectively, one year after surgery (Lee et al., 2011b). Diabetes remission corresponds with the significantly decreased homeostasis model of assessment-insulin resistance and C-peptide levels in obese patients with T2D after bariatric surgery (Lee et al., 2013; Chen et al., 2016). C-peptide is considered as a marker for pancreatic insulin secretion (Bonser & Garcia-Webb, 1984; Hovorka & Jones, 1994), and it is typically used as an index of beta-cell function (Berger, Stenström & Sundkvist, 2000). C-peptide is also one part of the ABCD score, which predicts the success of bariatric surgery for diabetes remission (Lee et al., 2013). Previous studies have been shown that insulin in the brain suppresses food intake in rats (Chen et al., 2012), and intranasal insulin administration reduces the human brain activity stimulated by pictures of food (Guthoff et al., 2010), intensifies satiety and reduces the intake of palatable snacks in women (Hallschmid et al., 2012), and enhances postprandial thermogenesis in healthy men (Benedict et al., 2011). In addition, incremental increases in the AUC for plasma insulin during an oral glucose tolerance test have been shown to predict lower food intake, lower carbohydrate consumption, and reduced weight gain in healthy Pima Indians (He et al., 2011). Collectively, the evidence indicates a role for insulin as a negative feedback signal in the regulation of energy intake and body weight. In our current study, post-operative C-peptide levels in patients after GB were negatively correlated with hunger and desire to eat. Moreover, post-operative C-peptide levels in the SG group were negatively correlated with hunger and fullness.

Lastly, a pleiotropic endocrine response and newly established jejunal nutrient-sensing may contribute to appetite reduction, as well as to long-term improvement in body weight and glycemic control after bariatric surgery. Altered secretions of gut hormones caused by anatomic rearrangement of the gastrointestinal tract after bariatric surgery have been proposed as one of the mechanisms underlying this phenomenon (Chen et al., 2009; Chen et al., 2010; Lee et al., 2011a; Ting et al., 2016). Recently, alteration of gut nutrient-sensing has been considered a potential therapeutic strategy to improve insulin sensitivity and glycemic control in diabetic rats after surgery (Breen et al., 2012; Ren et al., 2015). Collective evidence with our current results corroborates the concept that eating center controlling the consumption behaviors and appetite sensations is structured in a complex manner in the brain. However, these mechanisms require further investigation.

## Conclusions

In summary, T2D patients after either GB or SG exhibit distinct nutrient-induced consumption behaviors and appetite sensations post-operatively, which may account for the differential effects on weight loss and glycemic control after different surgery. Post-operative C-peptide levels have differential correlations with various appetite sensations in T2D patients after either GB or SG.

##  Supplemental Information

10.7717/peerj.3090/supp-1 Data S1 Raw data

## Additional Information and Declarations

### Data Availability

The following information was supplied regarding data availability: The raw data has been supplied as a Data S1.
